# Childbirth Readiness Scale (CRS): instrument development and psychometric properties

**DOI:** 10.1186/s12884-022-04574-6

**Published:** 2022-03-27

**Authors:** Yuan Mengmei, Zhao Meizhen, Zeng Tieying, Wu Meiliyang, Chen Ye, Zhang Ke, Tu AiQing

**Affiliations:** 1 Department of Nursing, Tongji Hospital, Tongji Medical College, Huazhong University of Science and Technology, 13 Hangkong Road, Wuhan, 430030 China; 2 School of Nursing, Tongji Medical College, Huazhong University of Science and Technology, Wuhan, China

**Keywords:** Childbirth readiness, Factor analysis, Pregnant women, Psychometrics properties

## Abstract

**Background:**

Childbirth preparation plays an important role in reducing maternal mortality and improving women’s childbirth experience. Evaluating childbirth readiness levels before and after interventions provides a basis for formulating more targeted and effective interventions. However, existing tools only assess partial childbirth preparation or have limited evidence of reliability and validity. The aim of this study was thus to develop a new instrument for use during the third trimester to comprehensively assess the readiness level of pregnant women, and test the scale’s psychometric properties.

**Methods:**

The scale was developed through exploratory mixed methods including qualitative and quantitative phases. A literature review and in-depth semi-structured interviews were utilized to identify the scale items. A Delphi expert consultation evaluated the content validity. Psychometric testing was conducted in a convenience sample of 731 pregnant women in the third trimester (recruited from 3 tertiary hospitals in Hubei province in China). Item analysis was used to screen items; exploratory factor analysis was performed to extract factors; confirmatory factor analysis was performed to evaluate fit on the factor structures.

**Results:**

The final scale consisted of four dimensions and 18 items that explained 65.8% of the total variance. Confirmative factor analysis (CFA) model showed that the 4-factor model fits the data well. The total Cronbach alpha coefficient of the total scale and 4 factors was 0.935 and 0.853–0.914. The split-half reliability was 0.880. The dimensions comprised “Self-management”, “Information literacy”, “Birth confidence” and “Birth plan”.

**Conclusions:**

The childbirth readiness scale we developed has good reliability and validity, and can be used to comprehensively assess the readiness level of pregnant women. In addition to understanding the overall level of women’s childbirth readiness, using subscale scores, improvements can be targeted to specific areas of the preparation for childbirth, to improve the efficiency of the intervention.

## Background

Pregnancy and childbirth are one of the most special and important experiences in a women’s life. It involves great challenges during pregnancy, including physical changes, psychological stress, role transformation, and family structure change [1]. Women also have to bear great physical pain during childbirth, and even face the threat of maternal and infant death. According to the latest World Health Organization (WHO) figures, about 295,000 women died during pregnancy and childbirth [2]. Childbirth is an important and multidimensional process with physical, psychological, emotional, social, and cultural dimensions. It is thus necessary to be fully prepared for the various aspects of all the links involved so that pregnant women can effectively cope with the challenges. Birth preparedness, as an important component of antenatal care called for by WHO, plays an important role in reducing maternal mortality and improving women's childbirth experience [3].

However, about 289,000 pregnant women in the world die of childbirth complications every year due to inadequate birth preparedness [4, 5]. Inadequate birth preparedness can endanger maternal and infant health and influence the childbirth outcome. Studies have shown that lack of birth preparedness may lead to preterm labour, prolonged labour, and increased risk of obstetrical complications and postpartum haemorrhage, increasing maternal mortality [6] The growth environment of the fetus would also be affected, resulting in adverse consequences such as low birth weight and neonatal asphyxia [7]. Moreover, poor preparedness can also lead to negative childbirth experiences, increasing the incidence of childbirth trauma, postpartum depression, and postpartum traumatic stress disorder (PTSD) [8]. And a further influence would be on women's desire for subsequent fertility, they may fear for the next pregnancy and not be willing to give birth again [9].

Given the serious influence of inadequate birth preparedness, at present, countries around the world have taken active interventions to help women prepare for birth [3, 10, 11]. Birth preparedness and complication readiness (BP/CR) is a strategy, widely used in low-resource settings countries and regions, to reduce delays in the use of skilled maternal and neonatal care, thereby reducing maternal and infant mortality [3]. Meanwhile, developed areas have developed rich prenatal preparation courses focusing on women's relevant knowledge for childbirth, self-care ability, and self-efficacy, to improve childbirth experience and satisfaction [10, 11]. However, most interventions are broad, uniform, and lack pertinence, causing great costs but little benefits. By assessing a women's level of readiness for childbirth, understanding where women are less prepared can provide the basis for more targeted and effective interventions. Knowing a women's readiness on the eve of childbirth may also help predict their ability to cope during labour. Therefore, it is important to measure women's level of readiness for childbirth and to have a practical instrument to assess it.

Several instruments have been developed to measure women's readiness for birth. The most widely used is the, developed by the maternal and neonatal health program [12]. This questionnaire was constructed based on the framework of BP/CR and focuses on physiological preparation and complications’ prevention. It contains 207 items in 12 dimensions, and due to a large number of items, many foreign scholars thus have adapted and simplified the questionnaire according to the local context and their study purpose and formed new assessment tools [13, 14], such as the Birth Preparedness Index (BPI) [15]. Notably, the Safe Motherhood questionnaire is widely used in low-resource settings countries and regions, while it is not applicable in high-resource countries and regions as most of the elements of BP/CR have already been met [16, 17]. Besides, indicators of the BP/CR framework focus on physiological aspects, emergency transport, financial preparation and skilled birth attendants, while other dimensions, such as psychosocial aspects, are neglected. Hudson etc. developed the Birth Preparedness and Assessment Index (BPAI), to measure women’s pre-pregnancy preparation, prenatal preparation, intrapartum preparation, and postpartum preparation [18]. However, these questionnaires lack good reliability and validity and ignore the importance of psychological preparation for childbirth. Carrie et al. developed the preparation for labour and birth instrument (P-LAB). It emphasizes the building of birth confidence in childbirth but lacks evaluation of other levels of preparation [19]. Therefore, there is currently a lack of a comprehensive tool to assess women's readiness for childbirth.

Birth preparedness plays an important role in reducing maternal mortality and improving women’s childbirth experience [3]. However, very little research has been conducted on birth preparedness in China. Due to the lack of a birth preparedness scale, some researchers have used specific scales to assess a certain aspect of birth readiness to predict women's overall level of childbirth readiness [20, 21]. Studies found that Chinese pregnant women's knowledge of birth preparedness, psychological readiness for childbirth, and birth planning were all at low levels [20, 21]. However, interventions of childbirth preparedness in China were mostly macro-control through relevant policies formulated by the government, which were broad, uniform, and lacked pertinence, resulting in a significant waste of costs and resources. Evaluating childbirth readiness levels before and after interventions could provide a basis for formulating more targeted and effective interventions. However, existing tools only assess partial childbirth preparation or have limited evidence of reliability and validity. The aim of this study is thus to develop a comprehensive assessment tool to evaluate the readiness level of pregnant women and test the scale’s psychometric properties.

## Methods

### Design

A methodological study of the exploratory mixed-method was implemented to develop the childbirth readiness scale (CRS). It included qualitative (generation an item pool and development of the CRS) and quantitative (assessment of psychometric prosperities of the CRS) phases. The setting for the study was three tertiary hospitals in Hubei, Wuhan, which is the megacity of central China. Two of the hospitals were general hospitals(Tongji Hospital Affiliated to Tongji Medical College of Huazhong University of Science and Technology, and the Third People’s Hospital of Hubei province) and one is a maternity and child health care center(Maternal and Child Health Care Hospital of Hubei province).

### Instrument development

To explain the concept and dimensions of childbirth readiness, a qualitative study was performed at the first stage. From July 2020 to October 2020, we conducted in-depth semi-structured interviews with pregnant women planning birth naturally, and prenatal care providers working for more than one year. We excluded women with a known severe mental illness that precluded participation in data collection, and women with medical problem (heart disease, diabetes, hypertension or renal disease for instance). A purposive sampling method was conducted to ensure sample diversity. Pregnant women varied in age, education level, parity, and gestational weeks; prenatal care providers varied in age, education level, working time, and job title. The principle for determining the sample size was practised as follows: after data saturation was reached, two more participants were interviewed. If no new topics were emerging, further recruitment would be terminated. With assistance from the head nurse in the maternity ward, the first author approached and invited potential participants, both pregnant women, and prenatal care providers. Consenting participants were required to sign an informed consent. In all, 24 pregnant women and 20 prenatal care providers were invited, and four pregnant women refused to participate. Two women said it was hard for her to talk about feelings and two did not report a reason for the refusal. Finally, 20 pregnant women and 20 obstetric medical staff were recruited for the study.

All interviews were conducted in Chinese by the first author who had been trained in conducting interviews. The interview time was set by the researcher in consultation with the interviewee. To eliminate unfamiliarity, the location of the interview was chosen to be in a private room of the maternity ward. Each interview lasted for 0.5–1.5 h. During the interview, when we found pregnant women were unprepared for information or psychological of childbirth, we would immediately give them verbal health education and distribute relevant brochures, and show our reassurance, encouragement, and support to them. After the interview, we would also inform their responsible doctors and nurses so that they can help in the follow-up treatment and care. All interviews were audio-recorded with participants’ consent and transcribed verbatim within 24 h. The records were then sent back to the participants to confirm accuracy. Content analysis was used to analyse qualitative data. The content analysis involved an overall reading of the transcripts, followed by a line-by-line initial coding. The initial coding was done independently by two researchers. The codes were classified into subcategories with similar meanings which were further grouped into main categories [22]. All data and field records obtained during the interview were recorded into Nvivo 10 to assist qualitative data analysis. Data transcription and analysis were also undertaken in Chinese, and only data and result presented in the writing publication were translated into English by the second author and checked by all authors.

The qualitative interview results showed that the preparation of the hospital bag for childbirth was the most mentioned by pregnant women. Secondly, many pregnant women felt that being psychologically prepared for birth was the most important thing. Some actively sought information about childbirth because they were concerned about their baby and their own health. The medical staff's concerns about childbirth preparation differed from those of pregnant women. They referred more to the physical aspects of childbirth preparation, such as regular antenatal examinations during pregnancy. Knowledge of pregnancy and childbirth was also stressed by them, especially knowledge of the process of childbirth, methods of analgesia, and postnatal self and newborn care. Content analysis led to the extraction of 4 themes including: 1-Self-management, 2-Information literacy, 3-Birth confidence and 4-Birth plan.

Subsequently, a literature retrieval was performed in PubMed, MEDLINE, CINAHL, EMBASE, Web of Science, and ProQuest. Key terms used in the search were “preparation”, “preparedness”, “readiness” combined with ‘pregnancy’, ‘perinatal’, ‘antenatal’, ‘antepartum’, ‘labour’, ‘birth’, ‘childbirth’. The search yielded 344 articles after removal of duplicates. After the title, abstract and full text review, 21 papers addressing preparation for childbirth were finally included. After comparing our extracted items and those in literature and omitting the duplicated items, the most relevant neglected items were selected to be added to the questionnaire to improve its comprehensiveness.

Based on the findings of the qualitative phase and literature review, an 82-item pool was created including 73 items from findings of the qualitative study and 9 items from the literature review. After reviewing the items, the research group developed a 38-item initial version of the CRS, consisting of four dimensions as “Self-management”, “Information literacy”, “Birth confidence” and “Birth plan”. A 5-point Likert scale was used for the initial scoring, which ranged from strongly disagree to strongly agree (on a scale of 1 to 5).

### Expert consultation and content validity

The Delphi method was used to reach a consensus among specialists. The number of specialists required for the Delphi method is not standardized and generally recommended to be between 15 to 50 [23, 24]. In this study, a total of twenty-four specialists were invited. They had from 10 to 50 years of experience in midwifery, obstetric clinical, obstetric nursing, nursing management, and nursing education. The specialists scored the importance of each item through a 5-point rating scale (5 = very important, 1 = not important). Meanwhile, they commented on the tool’s grammar, wording, item allocation, and scaling indices, and proposed suggestions to add, modify or remove items. The specialists were also asked to self-evaluate their basis for judging items and the degree of familiarity with the research content. The mean of importance and coefficient of variation (CV) of each item were calculated based on all the specialist responses. If an item obtained a mean score of importance less than 4 or a CV greater than 0.25, it was deleted [25]. The results showed that items’ mean importance ranged from 4.12 to 4.96, and the CV value ranged from 0.07 to 0.24, all met the standard.

At the same time, we evaluated the content validity. The item-level content validity index (I-CVI) and scale-level content validity index (S-CVI) were used to assess the content validity of the tool. The experts scored relevance of each item through a 4-point rating scale (4 = very much so, 1 = not at all). The I-CVI was calculated by dividing the number of experts with scores ≥ 3 by the total number of experts. The S-CVI was computed by calculating the mean CVI values. The S-CVI and I-CVI were calculated as 0.971(> 0.9) and 0.880–1.000(> 0.78), respectively. The results indicated that items of the scale had content validity [26].

Two rounds of Delphi expert consultation were conducted. In the first round, the research group discussed and revised the questionnaire dimensions and items according to the experts’ recommendations, and one item was deleted and seven items were added. The revised questionnaire was returned to the experts again for the second round of consultation. After collecting and analysing the second-round results, we combined two items and revised the statement of some items, and obtained a measurement with 4 dimensions and 43 items.

### Pilot testing

The preliminary scale was used in a pilot test conducted among a group of 30 pregnant women to examine the items’ readability and comprehensibility, survey structure, and item length. The pre-final version of the instrument had 43 items and was then processed for further psychometric testing.

### Psychometric properties test of the CRS

#### Sample

Convenient sampling was used in this study. The CRS was administered to pregnant women from 3 tertiary hospitals in Hubei province in China from November 2020 to February 2021. The inclusion criteria were: (a) in the third trimester; (b) planning a natural birth; (c) Being able to read and understand the scale explanations. The exclusion criteria were serious mental illness and history of major diseases such as cardiovascular diseases, diabetes, chronic. The sample size was estimated to satisfy the criteria of factor analysis [27]. Devillis suggests that proportions of 5 to 10 subjects to one variable is sufficient [28]. Because the draft CRS included 43 items, a sample size of 215 to 430 was estimated. Considering the 20.0% of sample loss rate, 269 to 538 participants were required.

#### Data collection

The data was collected face-to-face in the obstetrics clinic or ward. First of all, the pregnant woman would receive a consent form including the content and methods of this study, and standardized explanations of the research objectives and procedures. Those who agreed to participate completed questionnaires. In addition to the CRS, the questionnaires also included participants’ demographic characteristics including age, occupation, education level, residence status, gestational age, and parity. The questionnaires’ completion was checked by the researcher on the spot and the missing items were supplemented in time.

Sample 1 consisted of 365 pregnant women who were applied for item analysis as well as to assess the reliability and validity of the scale. Sample 2 consisted of 373 pregnant women who were applied to assess the scale’s degree of data fitting.

#### Data analysis

Item analysis was used to screen items. The construct validity of the tool was performed using both exploratory (EFA) and confirmatory factor analyses (CFA). On the other hand, the reliability was evaluated through internal consistency and stability of the instrument. EpiData3.1 data software was used for data management. All the statistical analyses and confirmatory factor analyses were performed using the SPSS version 25.0 and the AMOS version 24.0, respectively. *P* < 0.05 was taken as the level of statistical significance.

##### Item analysis

The items of the pre-final scale were screened by the critical ratio method, the correlation coefficient method, Cronbach’s α coefficient method, and the factor analysis method. The screening criteria are as follows:

a) The critical ratio (CR) method: Putting the scale’s score in order, the top 27.0% was the upper group, and the bottom 27.0% was the lower group. An independent samples t-test was used to compare the average score of each item between the two groups. The items whose CR value < 3 and *P* > 0.05 were deleted [29]; b) The correlation coefficient method: The item score was significantly correlated with the total score of the scale or the items with a correlation coefficient of 0.4 and above were retained [30]; c) Cronbach’s α coefficient method: If the Cronbach’s α coefficient increased after an item was deleted, the item was considered for deletion [31]; d) Factor analysis method: After factor analysis, item factor loading values < 0.5 were considered for deletion [32].

##### Construct validity

Exploratory factor analysis: The Kaiser–Meyer–Olkin (KMO) and Bartlett’s Test of Sphericity were used to test whether the sample was suitable for the factor analysis [33]. Maintaining the item required the factor loading ≥ 0.4 [34]. The decision on the factors extracted was based on the Scree plot and the eigenvalues above 1 [35].

Confirmative factor analysis: confirmative factor analysis was performed for comparing and assessing the model fitness. The model fit was checked using several fit indices including Chi-square and degrees of freedom ratio (χ ^2^/df), Root Mean Square Error of Approximation (RMSEA), Goodness of Fit Index (GFI), Comparative Fit Index (CFI), the Tucker Lewis Index (TLI), Normed Fit Index (NFI), Incremental Fit Index (IFI) and Relative Fit Index (RFI). Values of 3.0 or below for χ ^2^/df, values of 0.90 or more for GFI, CFI, TLI, and NFI, values of 0.08 or below for RMSEA, indicate acceptable model fit [36, 37].

##### Reliability

Reliability referred to the stability and consistency of the results measured by the questionnaire. Internal consistency of the subscales and the entire instrument was assessed by Cronbach’s alpha coefficient and split-half reliability. Generally, Cronbach’s alpha coefficient of 0.70 or above was considered acceptable, and 0.80 or more was recommended [38].

### Ethical considerations

The study received approval from the Ethics Committee of Tongji Hospital, Tongji Medical College, and Huazhong University of Science and technology (Code of Ethics approved: TJ-IRB20190614). All participants signed informed consent and were aware of the rights to withdraw their consent at any time without any penalty. All methods were carried out following relevant guidelines and regulations of the Committee of Tongji Hospital, Tongji Medical College, and Huazhong University of Science and technology.

## Results

### Participants

In all, 738 pregnant women were recruited in the study’s main survey. A total of 365 pregnant women in the third trimester were enrolled in the first round and 363 (99.5%) questionnaires were completed. A total of 373 pregnant women were enrolled in the second round and 368 (98.7%) questionnaires were completed and obtained. The mean age of all the participants was 29.84 years (SD = 3.366). The mean gestation was 36.24 weeks (SD = 2.085). The demographic characteristics of the two rounds of participants are shown in Table 1.

**Table 1:** Demographic Characteristics of participants (*N* = 731)

**Characteristics**		**Exploratory factor analysis (*n* = 363)**	**Confirmatory factor analysis (*n* = 368)**
Age (years)			
	Mean (SD)	29.74 (3.32)	29.95 (3.41)
	Range	18-41	22-44
Gestational age (weeks)			
	Mean (SD)	36.28 (2.13)	36.19 (2.04)
	Range	32-41	30-41
Parity		Number (%)	Number (%)
	Primipara	239 (65.8)	239 (64.9)
	Nulliparous	124 (34.2)	129 (35.1)
Highest Level of Education		Number (%)	Number (%)
	Junior high school and below	15 (4.1)	13 (3.5)
	High school	34 (9.4)	31 (8.4)
	College/university	244 (67.2)	255 (69.3)
	Postgraduate	70 (19.3)	69 (18.8)
Household Income (RMB/ per month)		Number (%)	Number (%)
	≤ 3000	8 (2.2)	9 (2.4)
	3001–5000	69(19.0)	62 (16.8)
	5001–10,000	144 (39.7)	157 (42.7)
	10,001–20,000	107 (29.5)	117 (31.8)
	> 20,000	35 (9.6)	23 (6.3)
Hospitalization		Number (%)	Number (%)
	Yes	338 (93.1)	353 (95.9)
	No	25 (6.9)	15 (4.1)
Living situation		Number (%)	Number (%)
	Live alone	3 (0.8)	5 (1.4)
	Live with partner	152 (41.9)	132 (35.9)
	Live with parents	28 (7.7)	34 (9.2)
	Live with partner and parents	180 (49.6)	197 (53.5)
Obstetric complications		Number (%)	Number (%)
	Yes	44 (12.1)	42 (11.4)
	No	319 (87.9)	326 (88.6)

*Note*. *SD* Standard deviations

### Item analysis

In Critical Ration, all items were statistically significant (*P* < 0.05). The correlation coefficient between items and the scale score was 0.43- 0.734 and all met the standard. When item 3 (I avoid bad lifestyles during pregnancy) was deleted, Cronbach's α coefficient increased. Besides, all items met the requirement of factor load ≥ 0.5, except for item 3. To sum up, item 3 was deleted (see Table 2).

**Table 2:** Item statistics results of childbirth readiness scale (43 items, *n* = 363)

Item	Critical Ration	Correlation Coefficient	Cronbach’s alpha if item deleted	Factor loading	Result
	**t**				
1	-12.725^***^	0.567^***^	0.969	0.56	keep
2	-16.634^***^	0.627^***^	0.969	0.63	keep
3	-8.027^***^	0.431^***^	0.970	0.43	delete
4	-10.161^***^	0.479^***^	0.969	0.49	keep
5	-12.607^***^	0.629^***^	0.969	0.63	keep
6	-11.928^***^	0.521^***^	0.969	0.54	keep
7	-14.719^***^	0.562^***^	0.969	0.58	keep
8	-16.322^***^	0.584^***^	0.969	0.60	keep
9	-12.075^***^	0.600^***^	0.969	0.59	keep
10	-15.745^***^	0.607^***^	0.969	0.63	keep
11	-15.526^***^	0.689^***^	0.968	0.68	keep
12	-17.285^***^	0.733^***^	0.968	0.72	keep
13	-15.347^***^	0.725^***^	0.968	0.71	keep
14	-13.528^***^	0.699^***^	0.968	0.68	keep
15	-13.403^***^	0.717^***^	0.968	0.69	keep
16	-15.551^***^	0.708^***^	0.968	0.69	keep
17	-12.674^***^	0.663^***^	0.969	0.64	keep
18	-14.666^***^	0.595^***^	0.969	0.62	keep
19	-14.191^***^	0.707^***^	0.968	0.69	keep
20	-14.265^***^	0.704^***^	0.968	0.69	keep
21	-14.603^***^	0.710^***^	0.968	0.70	keep
22	-13.349^***^	0.566^***^	0.969	0.58	keep
23	-19.423^***^	0.708^***^	0.968	0.72	keep
24	-16.130^***^	0.710^***^	0.968	0.70	keep
25	-12.157^***^	0.638^***^	0.969	0.63	keep
26	-17.085^***^	0.700^***^	0.968	0.71	keep
27	-17.130^***^	0.721^***^	0.968	0.73	keep
28	-18.517^***^	0.734^***^	0.968	0.75	keep
29	-13.177^***^	0.657^***^	0.969	0.65	keep
30	-12.385^***^	0.646^***^	0.969	0.64	keep
31	-13.396^***^	0.591^***^	0.969	0.61	keep
32	-16.130^***^	0.710^***^	0.968	0.72	keep
33	-15.994^***^	0.707^***^	0.968	0.71	keep
34	-18.396^***^	0.749^***^	0.968	0.77	keep
35	-20.081^***^	0.762^***^	0.968	0.78	keep
36	-16.577^***^	0.724^***^	0.968	0.73	keep
37	-20.170^***^	0.704^***^	0.968	0.72	keep
38	-17.476^***^	0.743^***^	0.968	0.74	keep
39	-17.982^***^	0.765^***^	0.968	0.76	keep
40	-14.251^***^	0.714^***^	0.968	0.70	keep
41	-13.307^***^	0.666^***^	0.969	0.66	keep
42	-17.283^***^	0.722^***^	0.968	0.73	keep
43	-20.302^***^	0.714^***^	0.968	0.73	keep

*Note*. ^***^ *P* < 0.001

### Exploratory factor analysis

Exploratory factor analysis was performed to evaluate the validity of the instrument structure. KMO and Bartlett’s tests were conducted before the EFA. The Kaiser–Meyer–Olkin was 0.934, and Bartlett’s Test of Sphericity was significant (chi-square = 4652.455, *p* < 0.0001) indicating samples' suitable for factor analysis. We conducted the first exploratory factor analysis on the 42 items of the pre-final version of CRS using Maximum Likelihood analysis (maximum variance orthogonal rotation). Without limiting the number of factors, five common factors with eigenvalues greater than 1 were generated, together explained 61.4% of the total variance. After analysis of the items contained in the five common factors, ten cross-loaded on multiple factors and five items with low factor loadings were excluded, for instance, item 31 (I can accept that baby’s sex is not as expected.) cross-loaded on factor3 and factor4; item 7 (When necessary, I take supplements as prescribed by my doctor during pregnancy) had a low factor loading on factor1, leaving 27 entries remaining.

Four common factors with eigenvalues greater than 1 were generated both in the second and the third exploratory factor analysis, together explaining 64.8% and 65.3% of the total variance, respectively. After analysis of the items contained in the four common factors, five cross-loaded on multiple factors and one item with low factor loadings were excluded in the second exploratory factor analysis, and three cross-loaded on multiple factors were excluded in the third exploratory factor analysis, for instance, item 29 (I can accept that the vaginal trial delivery may fail) had a low factor loading on factor3; item5 (I regulate my emotions during pregnancy) cross-loaded on factor1 and factor3, whereby the tool was reduced to 18 items.

**Fig. 1:**
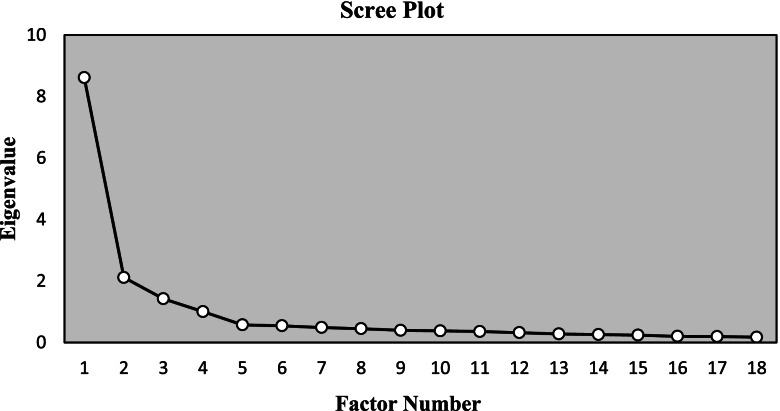
Scree plot and eigenvalue of exploratory factor analysis

We conducted a fourth EFA of the remaining 18 items. The scree plot showed four data points had an eigenvalue greater than 1.0, indicating that four dimensions were captured by the 18 items on the scale (Fig. 1). The first eigenvalue was 8.613, the second was 2.115, the third was 1.421, and the fourth was 1.004, which together explained 65.8% of the total variance. There is no item with cross-loaded or low factor loadings. The structure of the four-factor scale was generally consistent with the hypothetical ones. Each item was assigned under the original factor, so the names of the factors remain as before. A final 18-item tool loaded on 4 factors constructs as follows: Self-management (4 items), Information literacy (6 items), Birth confidence (4 items), and Birth plan (4 items) (Table 3).

**Table 3:** Factorial load matrix of exploratory factor analysis (*n* = 363)

Items	Factor loading			
	**F1**	**F2**	**F3**	**F**4
q1. I have regular antenatal examinations during pregnancy	0.489			
q2. I take care of my hygiene during pregnancy (e.g., vulvar skin care, oral care, breast care)	0.820			
q3. When necessary, I take medication as prescribed by my doctor during pregnancy	0.817			
q4. I will go to the hospital in time when I feel unwell during pregnancy (e.g., vaginal bleeding, abdominal pain, abnormal fetal movements)	0.701			
q5. I know about the process of natural childbirth		0.700		
q6. I know about the methods of labour analgesia (drug analgesia, non-drug analgesia)		0.804		
q7. I know how to cooperate with the medical staff during labour (e.g., body positions, force method)		0.824		
q8. I know about neonatal care (e.g., breastfeeding, umbilical care, skincare)		0.812		
q9. I know about postpartum self-care (e.g., diet, emotional regulation, common postpartum discomforts)		0.739		
q10. I know whom to ask if I have questions about childbirth		0.595		
q11. I believe I can bear the labour pain			0.656	
q12. I can insist on natural childbirth as long as conditions allow (including my own and my baby's condition)			0.739	
q13. I believe I can go through the delivery successfully			0.826	
q14. I am confident that I can cooperate with the medical staff to deal with emergencies during childbirth			0.721	
q15. I am prepared for the childbirth				0.746
q16. I have identified and learned about the hospital to give birth in advance				0.634
q17. I have decided my caregivers during childbirth				0.513
q18. I have decided how to feed my baby after childbirth				0.543

*Note*. F1: Self-management, F2: Information literacy, F3: Birth confidence, F4: Birth plan

### Confirmatory factor analysis

**Fig. 2:**
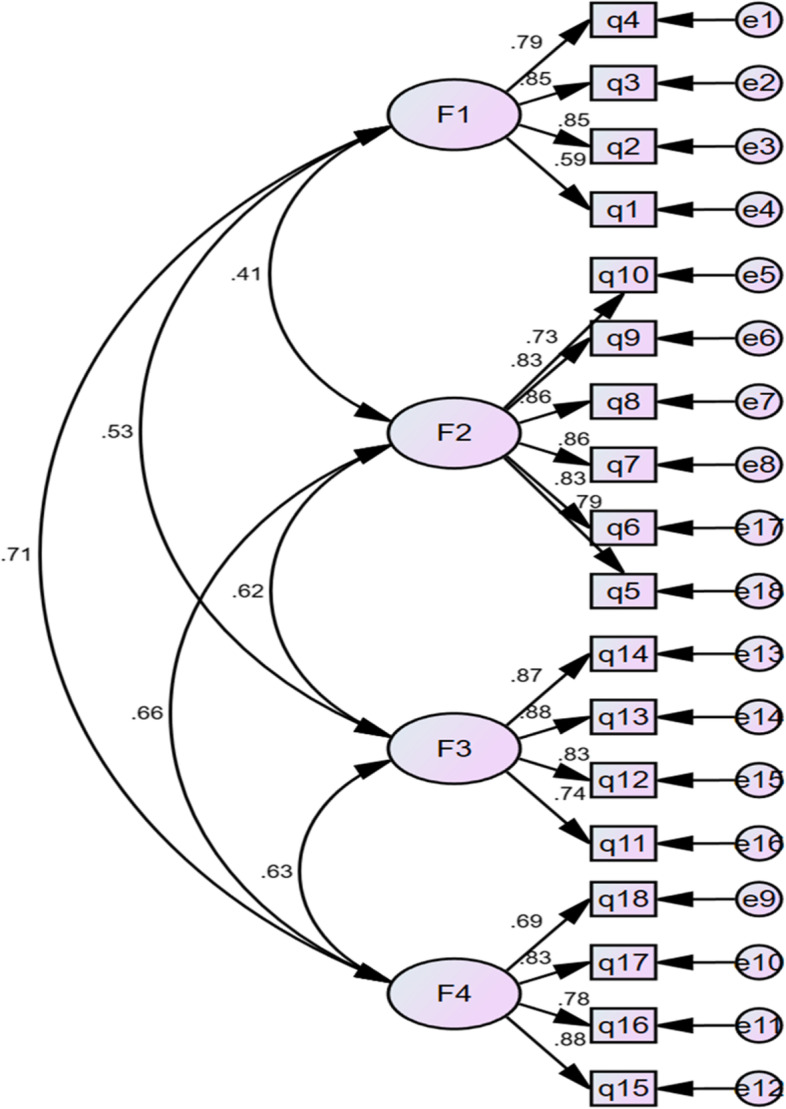
CFA standardised item loadings and factor correlations for Childbirth Readiness Scale (CRS; *n* = 368; *p* < 0.001). F1: Self-management; F2: Information literacy; F3: Birth confidence; F4: Birth plan

The 18-item questionnaire was subjected to confirmatory factor analysis to determine a model with appropriate fitness. The pattern was revised several times and an optimal pattern was eventually fitted and confirmed, as presented in Fig. 2. The value of χ ^2^/df was equal to 2.474, indicating the fitness of the model. The RMSEA of the model was equal to 0.063, falling in the acceptable range. The GFI, CFI, TLI, NFI, RFI, and IFI were more than 0.9, all indicating the 4-factor model fitted the data well (Table 4). The standardized regression coefficients of 18 items ranged from 0.59 to 0.88, above 0.5. In conclusion, the results of confirmatory factor analysis supported the structure of the four-factor model.

**Table 4:** Appropriate indices of model for CFA (*n* = 368)

Absolute Fit Indexes	result	Incremental Fit Indexes	result	Simplicial Fit Indexes	result
χ ^2^/df	2.474	NFI	0.932	PGFI	0.688
RMR	0.03	RFI	0.915	PNFI	0.785
RMSEA	0.063	IFI	0.958	PCFI	0.805
GFI	0.902	TLI	0.95		
AGFI	0.872	CFI	0.958		

*Note*. *χ *^*2*^ Chi-square goodness of fit statistic, *df* Degrees of freedom, *RMR* Square root mean residual, *RMSEA* Root-mean-square Error of Approximation, *GFI* Goodness-of-fit Index, *AGFI* Adjusted goodness-of-fit index, *NFI* Normed Fit Index, *RFI* Relative fit index, *IFI* Incremental fit index, *TLI* Tucker Lewis Index, *CFI* Comparative Fit Index, *PGFI* Parsimonious goodness-of-fit Index, *PNFI* Parsimonious normed Fit Index, *PCFI* Parsimonious comparative Fit Index

### Reliability

The internal consistency of the tool was assessed by Cronbach’s coefficient alpha. The Cronbach’s alpha and split-half reliability for the entire instrument was 0.935 and 0.880, and the coefficients for the subscales were 0.853 to 0.914, which showed proper internal consistency.

After evaluation of the validity and reliability, the final CRS was developed with 18 items and 4 dimensions including Self-management (4 items), Information literacy (6 items), Birth confidence (4 items), and Birth plan (4 items).

## Discussion

This study developed a new instrument, the childbirth readiness scale (CRS), through exploratory mixed methods including qualitative and quantitative phases. The tool was developed to comprehensively evaluate women’s readiness for childbirth. After a rigorous process of item generation and psychometric testing, the final CRS with 4 dimensions and 18 items using a 5-point Likert-type scale was formed and demonstrated construct validity, as well as good internal consistency. The four dimensions were “self-management”, “information literacy”, “birth confidence” and “birth plan”.

"Self-management" referred to the participation of a pregnant woman in part preventive or therapeutic healthcare activities, with the assistance of healthcare providers. In this study, the factor involved pregnant women's daily life behaviour, self-protection behaviour, and compliance behaviour. Self-management during pregnancy was a crucial part of birth preparedness since it could help women maintain a better physical and mental state for childbirth and improve pregnancy outcomes [39, 40]. Previous studies also proved that good self-management during pregnancy could effectively prevent and control health risk factors, reduce pregnancy complications and reduce the incidence of adverse pregnancy [41].

Factor two, "Information literacy", had six items to assess pregnant women's ability to acquire information and their knowledge of information about childbirth and postpartum. Pregnancy information was not included since it could be embodied through the factor “self-management”. The importance of knowledge about pregnancy and childbirth in birth preparedness has been highlighted in many recent studies [42, 43]. Improving the maternal and infant health literacy level of pregnant women was of great significance for preventing and reducing the occurrence of pregnancy-related diseases and adverse pregnancy outcomes [44]. Besides, studies have shown that information support can promote natural delivery and enhance the confidence of vaginal delivery, as sufficient information about childbirth can reduce fear of childbirth, enhance women's self-efficacy and their sense of control over childbirth [45, 46].

The third factor, "Birth Confidence" referred to a pregnant women's confidence or belief in her ability for natural childbirth. Many studies have shown that the psychological factors of pregnant women have a direct impact on childbirth. Birth Confidence has been associated with numerous positive consequences [17]. Studies have shown that childbirth confidence can help pregnant women feel prepared and increased satisfaction and positive birth experiences, and empowerment after birth [47, 48]. Women's childbirth self-efficacy beliefs relate to aspects of well-being during the third trimester of pregnancy [49]. Wan-Yim [50] found that self-efficacy can significantly reduce prenatal anxiety, improve the ability to cope with childbirth, and decrease labour pain.

The last factor of the CRS is the "Birth plan", which referred to women’s planning of childbirth experience during pregnancy, and expressed their expectations and needs of the birth process. It was an effective means of two-way communication between pregnant women and healthcare providers, and facilitated the establishment of a trust relationship between them, thus improving pregnant women's satisfaction with childbirth [51]. Whitford [52] strongly recommended the birth plan and believed it represented vitality and expectation, and the guarantee of a personalized service field to promote the health of the mother and child. Studies have demonstrated that the birth plan could positively influence patient satisfaction with birth, and reduce fear of birth and concern about the newborn’s health [53, 54]. A qualitative study found that most women considered the birth plan to be significant to their pregnancy and childbirth, even if the plan was not fully implemented [52].

The development of the CRS abided by the scientific process, and the tool had a beneficial and robust strong reliability and validity. First, the connotation of childbirth readiness was explored from different perspectives through semi-structured in-depth interviews with pregnant women and obstetric medical staff. Based on the results of qualitative research and literature retrieval, the research group preliminarily constructed the item pool. This ensured the applicability and comprehensiveness of the items. Then, two rounds of expert consultation were conducted to further ensure the objectivity, representativeness, and reliability of the items. The CRS had good content validity, indicating that the scale’s items can reflect the content to be measured. Besides, we used a variety of item analysis methods to screen the items, to ensure the items with great differentiation and homogeneity. Factor analysis showed that the CRS is a multidimensional scale composed of 4 factors, which was consistent with the results of the qualitative study and Delphi method analysis. The cumulative variance contribution rate of the four factors was 65.8%, and the item factor loading was ≥ 0.4, which reached the measurement standard. Confirmatory factor analysis showed that the parameters of model fitting degree reached ideal values, indicating that the scale had good structural validity. The internal consistency and split-half reliability of the CRS and each dimension was > 0.8, meeting the criteria for psychological instrument development, indicating that the CRS had good homogeneity and internal consistency and can be considered a good assessment scale.

The CRS scale developed in our study contains four domains, which shows that good self-management during pregnancy, knowing childbirth-related knowledge, building confidence for birth, and making birth plans are important aspects of childbirth preparation. This finding is consistent with previous studies, which have proved that these four factors could affect prenatal anxiety and fear, the birth outcome, the experience and satisfaction of childbirth, and the ability to cope with childbirth [19, 41, 44, 54]. This tool could be more applicable in China compared to other childbirth preparation assessment tools like BP/CR framework because it has been developed in line with the Chinese context. On the one hand, this scale can be used to comprehensively assess whether women are ready for childbirth, to understand the current status of women's preparedness for childbirth in China. Further, using subscale scores, improvements in policies and programs can be targeted to specific areas of the preparation for childbirth, to improve the efficiency of the intervention. It may be used to predict in advance a woman's ability to cope during childbirth. The study utilized psychometric recommendations, which helped to produce a tool that has content and construct validity and good internal consistency. This work can pave the way for other researchers to further carry out research related to childbirth preparation.

The limitations of this study include the following aspects: The convenience sample in this study were recruited from one geographic area and was limited to pregnant women presenting for care in the maternity clinic of tertiary hospitals. Therefore, future research can re-evaluate the instrument at different levels of hospitals. This study only represents pregnant women who are able to read and write and plan to have a natural birth, and lacks the representativeness for those with who are not able to read and plan to have the caesarean section. Besides, the instrument was developed in the context of Chinese culture it may not be transferrable to other populations. To use this tool, additional studies need to be conducted in larger populations and in different settings.

## Conclusion

The CRS is a new self-report instrument that can be applied to comprehensively assess women’s readiness for childbirth. Following a rigorous process of development and psychometric testing, the CRS has been shown to demonstrate good construct validity and internal consistency reliability. This valid and reliable instrument will be useful to help healthcare providers understanding the overall level of women’s childbirth readiness. Besides, using subscale scores, improvements can be targeted to specific areas of the preparation for childbirth, to improve the efficiency of the intervention.

## Data Availability

The datasets generated and/or analysed during the current study are not publicly available due to them containing information that could compromise research participant consent but are available from the corresponding author on reasonable request.

## Abbreviations

WHO: World Health Organization
PTSD: Postpartum traumatic stress disorder
BP/CR: Preparedness and Complication Readiness
BPI: Birth Preparedness Index
P-LAB: Preparation for Labour and Birth instrument
BPAI: Birth Preparedness and Assessment Index
CRS: Childbirth Readiness Scale
SD: Standard deviations
CV: Coefficient of variation
I-CVI: Item-level content validity index
S-CVI: Scale-level content validity index
CR: Critical ratio; KMO: Kaiser–Meyer–Olkin
EFA: Exploratory Factor Analysis
χ 2: Chi-square goodness of fit statistic
df: Degrees of freedom
RMR: Square root mean residual
RMSEA: Root-mean-square Error of Approximation
GFI: Goodness-of-fit Index
AGFI: Adjusted goodness-of-fit index
NFI: Normed Fit Index; RFI: Relative fit index
IFI: Incremental fit index
TLI: Tucker Lewis Index
CFI: Comparative Fit Index
PGFI: Parsimonious goodness-of-fit Index
PNFI: Parsimonious normed Fit Index
PCFI: Parsimonious comparative Fit Index
